# Reimagining Nodal Staging in Colorectal Cancer: Toward a Novel Non-Invasive Imaging Approach

**DOI:** 10.3390/cancers18132139

**Published:** 2026-07-02

**Authors:** Perla Moreno, Michela Orsi, Karl-Philippe Beaudet, Rania Benyahya, Leonardo Sosa-Valencia, Stéphane Cotin, Alfonso Lapergola, Alain García Vázquez

**Affiliations:** 1 IHU, Institute of Image-Guided Surgery, 1 Pl. de l’Hôpital, 67000 Strasbourg, Francealain.garcia@ihu-strasbourg.eu (A.G.V.); 2 Department of Surgical Sciences, University of Rome Tor Vergata, 00133 Rome, Italy; 3 Équipe MIMESIS, Inria, 2 Rue Marie Hamm, 67000 Strasbourg, France; 4 Maïeutique et Sciences de la Santé, Faculté de Médecine, Université de Strasbourg, 67000 Strasbourg, France; 5 Department of General Surgery, Nouvel Hôpital Civil, Université de Strasbourg, 1 pl. de l’Hôpital, 67000 Strasbourg, France

**Keywords:** lymph node metastasis, nodal staging, colorectal cancer, tumor biology, high frequency ultrasound, artificial intelligence, technological innovation

## Abstract

Colorectal cancer (CRC) remains the third most common malignancy worldwide and a leading cause of cancer mortality, largely driven by metastatic dissemination. Among metastatic routes, lymphatic spread is crucial to determine the prognosis and establish an adequate therapeutic strategy. Lymph node metastasis (LNM) defines stage III disease in the TNM classification, guiding adjuvant chemotherapy and surgical planning. However, nodal staging based on lymphadenectomy and histopathology is invasive, time-consuming, and may lead to overtreatment. Conventional imaging modalities, including computed tomography, magnetic resonance imaging, and endorectal ultrasound, show limited sensitivity and specificity for small or micro-metastatic nodes. Despite multimodal progress, no non-invasive technique reliably identifies malignant nodes in real time. PET–MRI, contrast-enhanced ultrasound, photoacoustic and fluorescence approaches, ICG mapping, and sentinel node biopsy improve detection but remain limited by specificity, cost, or availability. Extranodal extension (ENE) and tumor deposits (TDs) carry major prognostic value, reflecting aggressive biology and association with distant spread. Meanwhile, phylogenetic studies challenge linear dissemination models, indicating that some metastases arise directly from the primary tumor or TDs rather than LNMs. These data support refinement of staging and surgical strategies according to tumor biology rather than purely anatomical criteria. High-frequency quantitative ultrasound (HF-QUS) enables real-time, operator-independent, three-dimensional nodal assessment with reported sensitivity and specificity exceeding 85%. Combined with artificial intelligence and molecular profiling, it may support biologically informed staging, reduce unnecessary surgery, and foster precision oncology. Lymphatic dissemination in CRC offers a platform to merge tumor biology with technological innovation, where advanced imaging, molecular insight, and artificial intelligence may redefine nodal staging toward precision, non-invasive care.

## Simple Summary

Colorectal cancer is one of the most common cancers worldwide, and its spread to lymph nodes is a key factor that determines patient prognosis and treatment choices. However, current methods used to detect lymph node metastases, including imaging techniques and surgical removal followed by pathological examination, are often invasive, costly, and not always accurate, especially for small or early metastatic deposits. As a result, some patients may therefore undergo unnecessary surgery, while others may not receive the most appropriate treatment on time. This review summarizes current knowledge on how colorectal cancer spreads through the lymphatic system and evaluates the strengths and limitations of existing diagnostic approaches. It also discusses emerging technologies, such as advanced ultrasound techniques and artificial intelligence, which may allow more accurate, non-invasive, and real-time assessment of lymph node involvement. These innovations could improve staging accuracy and support more personalized treatment strategies in the future.

## 1. Introduction

Colorectal cancer (CRC) is the third most common cancer worldwide, with a significant mortality rate linked to metastatic progression. Tumor dissemination to the lymph nodes (LNs) represents a key step in the progression and prognosis of CRC [1]. Indeed, lymph node involvement is a fundamental criterion for TNM staging system, which guides therapeutic decision making [2].

Current assessment of lymph node metastasis (LNM) in colorectal cancer primarily relies on surgical resection and histopathological evaluation, which can delay therapeutic decisions and expose patients to potentially unnecessary procedures and risks. Furthermore, conventional imaging modalities frequently exhibit limited sensitivity and specificity in the detection of nodal metastases, thereby constraining their reliability in preoperative staging. Consequently, there is an urgent need for a non-invasive, accurate, and reproducible imaging strategy capable of reliably assessing nodal status in CRC, providing not only anatomical, but also biological characterization. The implementation of a non-invasive imaging approach, combined with an enhanced understanding of the mechanisms underlying tumor cell dissemination to the lymphatic system, has the potential to confer substantial clinical and economic benefits. By reducing dependence on invasive procedures, this strategy may lower procedure-related complications, enable more targeted delivery of adjuvant therapies, shorten hospital stays, and optimize healthcare resource utilization, thereby supporting a more efficient and sustainable framework for colorectal cancer management.

## 2. Relevant Section

### 2.1. Cellular and Molecular Mechanisms of Lymphatic Metastasis

LNs function as critical hubs for metastatic tumor cell growth, secondary dissemination to other tissues, and modulation of antitumor immune responses [3]. The presence of LNM significantly impacts cancer staging, clinical management, and prognosis, making it a pivotal consideration in patient evaluation [4]. Lymphatic metastasis in CRC is a multi-step process in which tumor cells acquire invasive and migratory properties. Recent evidence highlights complex metastatic seeding patterns in CRC, where both primary-to-metastasis and metastasis-to-metastasis spread contribute substantially to disease progression [5].

To properly evaluate this biological landscape of lymphatic dissemination, it is necessary to distinguish well-established clinicopathological drivers from emerging, speculative biomolecular pathways. While factors regulating angiogenesis and macro-environmental remodeling represent validated cornerstones of colorectal cancer progression, fine-scale cellular components—such as voltage-gated sodium channels (VGSCs), specific aquaporins, and localized extracellular matrix (ECM) remodeling factors—currently represent exploratory, preclinical findings that characterize the front-line of basic translational research rather than immediate diagnostic targets.

At the cellular level, the epithelial–mesenchymal transition (EMT) enables tumor cells to lose epithelial characteristics, such as polarity and tight cell–cell contacts, adopting invasive and migratory mesenchymal phenotypes that facilitate dissemination from the primary tumor site [6].

At the molecular level, proteins involved in ECM remodeling and cell adhesion, such as Heat Shock Protein 47 (HSP47) and insulin-like growth factor-1 receptor (IGF-1R), support cancer cell invasion into lymphatics by enhancing collagen deposition and cytoskeletal rearrangement [7,8]. Endothelial-specific adhesion molecule (ESAM) expression on endothelial cells promotes enhanced migration and tube formation, aiding lymphatic vessel formation [9,10]. Additionally, in preclinical models, voltage-gated sodium channels (VGSCs) have been shown to modulate cancer cell migration, invasion, and ECM degradation through pathways involving matrix metalloproteinase (MMP) activity, further facili-tating lymphatic dissemination [11,12]. Although the clinical mechanisms are still un-clear, molecules such as aquaporins (AQPs) represent an exploratory avenue of re-search into lymphatic metastasis, contributing to microenvironment alteration, mi-gration, invasion, and lymphangiogenesis [13].

Central to metastatic progression is lymphangiogenesis, the formation of new lymphatic vessels, which facilitates tumor spread by providing pathways for cancer cell migration and invasion. As such, increased lymphatic vessel density and intratumoral lymphangiogenesis have been observed in peritumoral tissues, providing conduits for tumor cells to access the lymphatic system [14].

Lymphangiogenesis is driven by a complex interplay of growth factors, cytokines, enzymes, bioactive lipids, chemokines, adhesion molecules, and noncoding RNAs. Vascular endothelial growth factor-C (VEGF-C) is among the most well-characterized factors, activating VEGF receptor-3 (VEGFR-3) on lymphatic endothelial cells (LECs) and triggering intracellular signaling cascades (PKC/ERK and AKT) that promote LEC proliferation and migration [2,15]. Beyond VEGF-C, other contributors include fibroblast growth factor-2 (FGF-2), platelet-derived growth factor-BB (PDGF-BB), insulin-like growth factor-1 (IGF-1), epidermal growth factor (EGF), hepatocyte growth factor (HGF), and various pro-inflammatory cytokines such as TNF-α, IL-6, IL-7, and IL-17 [16,17,18,19]. Lipid metabolism regulators, like fatty acid synthase (FASN), and signaling molecules, such as sphingosine-1-phosphate (S1P) and lysophosphatidic acid (LPA), also facilitate lymphangiogenesis and metastatic progression [20,21,22]. Adhesion molecules, including integrin α4β1 and CD146, play additional roles in LEC sprouting and migration [23,24].

Fluid dynamics also contribute substantially to LNM initiation. Tumor-associated abnormalities in blood vessel permeability and blood flow cause elevated interstitial fluid pressure (IFP) within tumors, which creates a pressure gradient that directs interstitial fluid—along with tumor cells and tumor-derived factors—into lymphatic vessels and towards draining LNs [25,26]. Accordingly, higher IFP levels have been correlated with metastatic progression in preclinical models [27].

Once tumor cells reach lymphatic circulation, their capacity to invade LNs is closely linked to the expression of specific receptor proteins and cytokines that enable evasion or suppression of local immune defenses, thereby fostering a permissive microenvironment for malignant cells [28].

A critical preparatory step for metastatic colonization involves the formation of a premetastatic niche (PMN) in LNs, which is shaped by tumor-derived soluble factors, extracellular vesicles (EVs), and tumor antigens that modulate the LN microenvironment to support disseminated tumor cell survival and proliferation [29,30]. This PMN establishment is characterized by lymphangiogenesis, secretion of immunosuppressive cytokines (IL-10, IL-13, IFN-γ), immune cell alterations, including dendritic cell and T cell impairment, high endothelial venule dilation, and extensive remodeling of the ECM with fibroblastic reticular cell hyperproliferation and accumulation of collagen and hyaluronic acid [31].

Upon reaching LNs, tumor cells adapt metabolically to the lipid-rich LN microenvironment by upregulating fatty acid oxidation (FAO) and activating peroxisome proliferator-activated receptor alpha (PPAR-α) pathways, allowing accumulation of fatty acids beyond levels found in primary tumors [32]. Immune evasion is also a hallmark of successful LN colonizers, commonly via downregulation of major histocompatibility complex (MHC) class I molecules to avoid T cell recognition [33,34,35]. The interaction between tumor cells and immune components within LNs often induces immune tolerance, enabling successful metastatic seeding within the niche [36].

Intricate cellular and molecular alterations shape metastatic microenvironments within LNs, promoting tumor survival, immune evasion, and further dissemination. These changes likely translate into subtle yet significant structural variations in metastatic lymph nodes that might hold the potential to be detected in recognizable image patterns by advanced modalities such as high-definition ultrasound, offering promising avenues for improved diagnostic precision (Table 1).

### 2.2. Clinical and Prognostic Implications of Lymphatic Dissemination

LN involvement remains a critical determinant of prognosis and treatment planning in CRC. The presence of LNM at the time of diagnosis indicates locoregional dissemination and significantly affects overall survival and recurrence-free survival, with a reported 28% reduction in five-year survival compared to patients without nodal involvement [37]. Despite its prognostic value, the therapeutic utility of lymphadenectomy remains controversial across various cancer types. For example, in early-stage breast cancer, axillary dissection has not demonstrated superiority over radiotherapy in improving survival among LN-negative patients [38], and similar conclusions have been drawn in melanoma and other malignancies [39]. Hence, there is a growing trend within the medical community toward more selective and conservative lymph node dissections, aiming to balance oncologic efficacy with reduced surgical morbidity.

In CRC, preoperative assessment of the risk of LNM serves two essential roles: it enables a more accurate prognosis and guides therapeutic strategy, particularly in the context of neoadjuvant treatment. LN status, defined by the TNM staging system, considers the number and location of metastatic lymph nodes. This staging delineates treatment pathways; patients with stage I–II (node-negative) disease may be treated with surgery alone, while those with stage III (node-positive) CRC typically receive adjuvant chemotherapy to reduce recurrence risk [40]. Furthermore, for patients with locally advanced disease, neoadjuvant therapy may be utilized to achieve tumor downstaging and facilitate resection.

The extent of nodal involvement quantified by histopathological parameters such as extranodal extension (ENE) and tumor deposits (TDs) adds further prognostic gran-ularity [41]. To optimize radical lymphadenectomy and overcome compliance barriers in nodal harvesting, near-infrared (NIR) fluorescence imaging utilizing Indocyanine Green (ICG) has emerged as a crucial intraoperative tool. ICG fluorescence imaging serves a dual purpose in colorectal oncological surgery: it allows for real-time assessment of tissue perfusion, ensuring adequate perfusion of bowel segments prior to anastomosis, and it provides high-contrast visualization of regional lymphatic path-ways. This fluorescence-guided mapping enhances the identification of sentinel lymph nodes and aberrant lymphatic basins, allowing a more tailored lymphadenectomy and ensuring the removal of an adequate number of nodes for precise staging [42]. Complementary to this, intraoperative sentinel lymph node mapping via subserosal ink injection downstream of the tumor continues to offer utility in detecting the pri-mary draining node through localized lymphatic channels, potentially reducing the need for blindly extended dissections in early-stage disease. ENE is characterized by tumor cell infiltration beyond the nodal capsule and correlates with poor prognosis [43]. TDs are defined as isolated malignant cell clusters in the mesocolic fat without re-sidual nodal, vascular, or neural structures and have been associated with worse out-comes than ENE [44]; as demonstrated in an international meta-analysis where TDs were useful predictors for the development of liver and lung metastases in colorectal cancer [45]. Both TDs and ENE reflect aggressive tumor biology, with some evidence suggesting that ENE may represent a transitional phase toward TD formation [45].

LNM has traditionally been considered the primary conduit for distant dissemination in CRC. Nevertheless, recent phylogenetic analyses challenge the traditional view upheld by the TNM staging system and demonstrate that distant metastases follow biologically distinct routes. A recent study demonstrated that liver metastases arose from TDs, LNM, the primary tumor, or a combination of these in 23%, 23%, 23%, and 30% of cases, respectively. Notably, the origins of peritoneal metastases showed a markedly different pattern; 40% were traced to TDs and 37% to the primary tumor, indicating a predilection for peritoneal spread via TDs rather than LNM (10%) [46]. These findings are consistent with another study, which used genetic analysis of tumor clones to investigate metastatic origin; it revealed that in 65% of cases, distant metastases arose from subclones that were genetically independent of those found in LNM, suggesting that LNM directly contributes to the development of distant metastases in only 13% to 20% of colorectal cancer cases [47].

The distinct evolutionary trajectories of liver and peritoneal metastases suggest that the current TNM framework may not fully capture the metastatic complexity in CRC. Both TDs and LNM were identified as potential sources of distant disease, but their relative contributions vary by metastatic site, highlighting the need to re-evaluate nodal staging paradigms considering this biological heterogeneity of metastatic seeding. Furthermore, embryonic origin and anatomical location dictate distinct lymphatic pathways and prognostic profiles between embryologically right-sided (midgut) and left-sided (hindgut) colon cancers. Right-sided tumors primarily drain along the supe-rior mesenteric vein axis and frequently exhibit distinct molecular profiles, such as microsatellite instability (MSI), which correlate with altered regional immune re-sponses. Conversely, left-sided tumors drain via the inferior mesenteric system. Rectal cancers present the highest anatomical complexity, where lymphatic dissemination can occur not only cephalad along the superior rectal vessels but also laterally toward the internal iliac and obturator nodes. This complex compartmentalization justifies a specific, topographically adapted surgical approach—such as Total Mesorectal Exci-sion (TME) and Complete Mesocolic Excision (CME)—to maximize lymph node recov-ery and limit local recurrences [48].

Altogether, these findings highlight the clinical and prognostic relevance of lymphatic dissemination evaluation in CRC. Both nodal and extranodal characteristics play a critical role in risk stratification and directly influence therapeutic decision-making. In this context, factors such as accurately determining the stage of nodal invasion, establishing the need and extent of lymphadenectomy, and the number of evaluated LNs during histopathological analysis gain substantial relevance.

### 2.3. Nodal Staging Pitfalls

#### 2.3.1. Controversies Regarding the Extent of Lymphadenectomy

With the implementation of mass screening for CRC, the detection of early-stage cancers is increasing. Nevertheless, the need for radical surgery, which allows assessment of nodal status, is a matter of debate among multidisciplinary teams, especially for very early cancers (pT1).

In the presence of high-risk histological features such as poor tumor differentiation or lymphatic invasion, additional surgery is often recommended. However, the limitations of current histological criteria have significant clinical implications. Using existing clinicopathological parameters, approximately 60–70% of patients with pT1 CRC are classified as high-risk. Yet, pathological examination of surgical specimens reveals LNM in only 2–10.5% of cases, and residual tumor in fewer than 20%. Consequently, more than 80% of patients may therefore undergo unnecessary surgery [49]. This discrepancy raises a critical question: is the systematic pursuit of LNM primarily a tool for staging and guiding adjuvant therapy, or does its surgical removal meaningfully contribute to preventing disease progression?

Notably, studies have demonstrated that more extensive nodal dissections are associated with improved survival, likely due to enhanced staging accuracy and more appropriate use of adjuvant therapy [50,51]. Therefore, adequate lymphadenectomy remains central to optimizing oncologic outcomes, and failure to remove or detect metastatic lymph nodes can lead to understaging, depriving some patients of appropriate adjuvant treatment. A consistent association has been reported between higher LN yields and better survival outcomes, in both node-negative and node-positive patients [52,53]. Still, a higher number of nodes examined does not necessarily correlate with more nodes being positive. To account for variability in nodal yield, the use of the LN ratio (number of positive LNs relative to the total examined) has emerged as a complementary measure to better stratify risk in stage III disease [41]. Surgical oncologic standards, including high vascular ligation, CME, and sufficient circumferential margins, must be complemented by diligent pathological examination. Guidelines from multiple expert bodies, including the American Joint Committee on Cancer and the College of American Pathologists, recommend evaluating at least 12 LNs to ensure accurate staging [54,55]. Yet, population-based data suggest that compliance remains suboptimal, with only 37% of patients receiving adequate nodal evaluation [56]. This variation can be attributed in part to pathology practice patterns and specimen-handling techniques [57,58], but many other factors might play an important role, such as surgeon experience and patient characteristics (e.g., obesity). During surgery for nodal staging, intraoperative complications are primarily related to the technical complexity of central and mesenteric lymphadenectomy. Significant hemorrhage may occur from injury to major mesenteric vessels or their branches, particularly during dissection along the superior mesenteric axis or at the root of the mesentery, and may necessitate advanced vascular control or conversion to open surgery. Extensive mesenteric mobilization increases the risk of bowel injury, devascularization, or ischemia due to disruption of the microvascular supply. Additional risks include thermal or traction-related damage to adjacent organs and lymphatic leakage from disrupted channels. Meticulous surgical technique, precise identification of vascular variants, and a thorough understanding of lymphatic anatomy are critical to minimizing these complications, which are reported in approximately 4–8% of lymphadenectomy procedures [59,60].

Therefore, nodal staging, which remains invasive and potentially associated with significant morbidity, must be supported by clear clinical benefit. Consequently, the ability to accurately identify LNM and related features through non-invasive imaging becomes increasingly essential.

As our understanding of tumor biology evolves and diagnostic technologies advance, it is likely that novel pathological and clinical indicators will be incorporated into an updated staging framework, allowing the identification of biologically relevant LNM that drive disease progression, offering a refined risk stratification and supporting personalized treatment strategies. Although these insights are not yet sufficient to warrant immediate changes to current clinical guidelines or the TNM staging system [55], they establish a critical foundation for innovative translational research and the future refinement of CRC management. In this context, accurate identification of LNM during the evaluation of patients with colorectal cancer remains of paramount importance.

#### 2.3.2. Current Challenges in Detecting Lymph Node Metastases

Preoperative LN staging in CRC remains a significant clinical challenge despite advances in imaging modalities. Small LNs, often less than 5 mm, can be difficult to detect using conventional methods, leading to a risk of underestimation [61].

As curative treatment strategies increasingly include advanced endoscopic approaches such as endoscopic submucosal dissection (ESD) and endoscopic full-thickness resection (EFTR), accurate preoperative staging has become even more critical in early-stage CRC. The incidence of T1 CRC treated endoscopically is rising [62,63], and histopathological evaluation remains the cornerstone for determining the risk of LNM. Therefore, clinical guidelines highlight the importance of endoscopic and histological assessment in guiding the decision between endoscopic and surgical resection, given the morbidity, costs, and quality-of-life impact associated with surgery [64,65,66].

Beyond LNM identification, accurate anatomic localization of nodal involvement is essential, as mesorectal and perirectal nodes represent locoregional disease (stage III), whereas involvement of retroperitoneal or common iliac nodes upstages CRC to stage IV. However, current computed tomography (CT) and magnetic resonance imaging (MRI) often struggle to differentiate these compartments preoperatively, leading to potential misclassification [67,68]. This highlights the urgent need for more reliable, noninvasive diagnostic strategies capable of improving nodal staging accuracy and reducing overtreatment in early-stage disease.

Advances in molecular imaging and digital pathology allow for more detailed analysis of LN tissue; machine-learning algorithms are increasingly being used to automate LN detection and classification. These algorithms could help standardize and improve the reproducibility of LNM detection to optimize the quality of staging and the patient’s treatment pathway.

#### 2.3.3. Preoperative Evaluation of Metastatic Lymph Nodes

Several non-invasive imaging techniques are available to confirm the presence of LNM. In this section, we will focus on ultrasound, abdominopelvic CT, MRI, positron emission tomography–computed tomography (PET–CT), and positron emission tomography–magnetic resonance imaging (PET–MRI).

Endorectal ultrasound (ERUS) is the main technique used for locoregional staging in CRC, especially for rectal tumors. It provides high-resolution images of the rectal wall and surrounding LNs, allowing for detailed assessment of nodal morphology. Nodes exceeding 5 mm in short axis, particularly if rounded, with cortical changes and absence of a central fatty hilum, are generally regarded as suspicious for metastatic involvement [69,70]. The presence of a heterogeneous or markedly hypoechoic cortex, central necrosis appearing as anechoic zones, and complete effacement of the hilum are morphological criteria based on the underlying structural changes associated with tumor infiltration. Moreover, Doppler ultrasound can detect abnormal peripheral blood flow around the node, suggesting tumor-induced angiogenesis [71]. However, the reliability of morphology-based assessment remains inherently limited. Reactive or inflammatory lymph nodes may also show enlargement, cortical thickening, and altered vascular patterns, leading to false-positive findings. Conversely, micrometastases may not produce detectable morphological changes, particularly in small lymph nodes, resulting in false negatives. Therefore, although morphological analysis provides valuable initial clues for identifying suspicious nodes, it lacks sufficient specificity and sensitivity when used in isolation. The sensitivity of ERUS to detect metastatic lymph nodes is commonly reported between 90 and 95%, and specificity between 70 and 90%, depending on operator skill and equipment quality [72]. However, ERUS is limited in visualizing deeper mesenteric or pelvic lymph nodes, so it is usually complemented by MRI or CT for comprehensive staging.

Abdominopelvic CT is commonly used to evaluate distant metastases and LN involvement beyond the pelvis. It provides good spatial resolution and rapid imaging over large anatomical areas, making it effective for detecting LNs larger than 10 mm in short-axis diameter, which are generally considered suspicious [73]. However, CT’s accuracy in distinguishing metastatic from reactive LN based on size alone is limited, resulting in moderate sensitivity ranging from 60 to 85% and specificity between 70 and 90% [74]. Morphological criteria such as irregular nodal borders, heterogeneous contrast enhancement, and central necrosis can improve diagnostic accuracy but are not fully reliable [75]. Because CT alone offers suboptimal accuracy for nodal staging, it is most often combined with MRI or ERUS to provide a more complete preoperative assessment.

MRI is widely used in CRC staging, especially for evaluating rectal tumors and regional LNs, due to its excellent soft-tissue contrast and multiplanar capabilities [76]. MRI identifies LNs suspicious for metastasis primarily based on size (larger than 5 mm in short-axis diameter), irregular borders, heterogeneous signal intensity, and the absence of a fatty hilum [77,78]. Advanced MRI techniques like diffusion-weighted imaging (DWI) improve detection by highlighting areas of restricted water diffusion, which often correspond to metastatic involvement. The sensitivity of MRI for nodal metastasis detection ranges from 70 to 85%, with specificity between 70 and 90%, depending on the imaging protocol and radiologist experience [79]. MRI also excels in assessing the mesorectal fascia involvement and depth of tumor invasion, critical for surgical planning. Despite its strengths, MRI can sometimes overstage nodes due to inflammation or reactive changes [76]. The reliability of MRI-based morphological and functional assessment remains imperfect, leading to false-positive findings. Conversely, small metastatic deposits or micrometastases may not significantly alter nodal morphology or diffusion characteristics, resulting in false negatives. In addition to its diagnostic limitations, MRI also presents practical constraints that may impact its widespread use in routine clinical practice. High costs, limited availability in certain healthcare settings, and longer acquisition times compared to CT can restrict accessibility. Variability in imaging protocols and interpretation across institutions may also affect reproducibility and diagnostic consistency.

While ERUS carries the risk of mild discomfort or minor bleeding, and CT and MRI have potential risks associated with contrast agents or radiation exposure, these complications remain relatively uncommon. In a comparative effectiveness review, MRI was found to be less likely to overstage CRC nodal status than CT, although no significant differences were observed in accuracy, overstaging, or understaging between MRI and ERUS. Overall, conventional methods such as CT, MRI, and ERUS demonstrate comparable overall accuracy but are limited by suboptimal sensitivity for nodal metastasis detection [80]. To overcome this concern, complementary imaging methods were added to the initial evaluation protocols.

PET–CT is used in CRC staging to detect metabolically active LNs and distant metastases by combining functional and anatomical imaging. PET–CT identifies suspicious LNs based on increased uptake of the radiotracer fluorodeoxyglucose (18F-FDG), which highlights areas of high metabolic activity typical of tumor cells [81]. This modality is particularly valuable for detecting distant metastatic disease and recurrent cancer, as it can reveal lesions not visible on CT or MRI alone [82]. Sensitivity for detecting metastases is influenced by tumor size and metabolic activity [83]; therefore, small LNM may be missed due to limited spatial resolution, and inflammatory or infectious processes, which increase metabolic activity and FDG uptake [84].

PET–MRI combines the metabolic imaging of PET with the soft tissue acquisition of MRI, offering enhanced detection and characterization of CRC lesions and LNM. This hybrid modality also provides better soft-tissue contrast for evaluating tumor invasion depth and surrounding structures, which is critical for treatment planning [85]. PET–MRI has several limitations, including longer acquisition times and limited availability [86].

Compared with 18F-FDG PET/CT, high-resolution pelvic MRI demonstrates superior sensitivity for identifying nodal metastases (61% vs. 94%), though PET/CT shows higher specificity (83% vs. 67%) [87]. Similarly, 18F-FDG PET/MRI has emerged as a promising technique, with pooled sensitivity and specificity of 0.81 and 0.89, respectively, suggesting improved diagnostic performance over PET/CT for nodal staging, although study heterogeneity remains a limitation [88]. Conversely, a meta-analysis of 18F-FDG PET/CT reported a sensitivity of only 42.9% and a specificity of 87.7%, demonstrating a limited role as a routine tool for CRC nodal staging [82] (Table 2).

Beyond conventional imaging, innovative approaches such as CT texture analysis and ultrasonographic texture analysis are being explored. These methods assess tissue heterogeneity, providing quantitative data beyond morphological criteria, and have shown promise in distinguishing between benign and metastatic LNs [89,90,91]. More recent developments, such as contrast-enhanced ultrasound with microbubble agents, offer dynamic and relatively inexpensive imaging, though their use is constrained by operator dependence and modest contrast resolution [92].

Emerging modalities also include lymphoscintigraphy, CT lymphography, and hybrid approaches such as photoacoustic or fluorescence imaging, which offer improved visualization of lymph nodes [93,94,95,96] (Table 3). However, limitations such as poor spatial resolution, radiation exposure, or high costs restrict their widespread adoption.

In selected contexts, endosonographic procedures have also been evaluated for restaging purposes. Data from mediastinal lymph node evaluation highlights the potential utility of such approaches. A systematic review and meta-analysis including 574 patients undergoing restaging with endobronchial ultrasound (EBUS) guided transbronchial needle aspiration (TBNA) or endoscopic ultrasound-guided fine-needle aspiration (EUS-FNA) demonstrated a pooled sensitivity and specificity of 67% and 99%, respectively [97]. Although extrapolated from lung cancer, these findings underline the promise of advanced imaging and sampling technologies for difficult restaging scenarios in CRC, especially in the setting of fibrosis or adhesions after neoadjuvant therapy.

It is critical to note that the diagnostic performance metrics reported across these mo-dalities—particularly sensitivity and specificity ranges—must be interpreted with cau-tion. Substantial inter-study variability exists due to significant heterogeneity in insti-tutional imaging protocols, magnet strengths for MRI, radiotracer uptake times in PET systems, and varying patient population characteristics (such as body mass index and tumor stage distribution). Consequently, these pooled values reflect overall diagnostic trends rather than absolute, standardized performance caps. Additionally, specific regional constraints must be acknowledged, such as the limited accuracy of PET/CT frameworks within the small pelvis, where physiological radiotracer accumulation in the bladder and pelvic organs frequently yields confounding false-positive or false-negative findings in close proximity to reproductive and genital structures.

#### 2.3.4. Intra-Operative and Sentinel Lymph-Node Biopsy Guidance Methods

Intra-operative LN assessment provides critical information and guides the following therapeutic decisions. Several intraoperative and sentinel lymph node (SLN) biopsy guidance methods have been developed, each with distinct strengths and limitations [98].

Fine-needle aspiration biopsy (FNAB) has historically been employed to assess axillary LNs. However, its diagnostic accuracy is limited, as FNAB has been reported to exhibit a false-negative rate ranging from 11 to 20% and a highly variable sample yield (0–53%) [99]. This variability reduces its reliability as a standalone staging tool.

Blue dye mapping remains one of the most widely used techniques, primarily due to its intuitive application and cost-effectiveness. Nevertheless, it demonstrates a low SLN identification ratio, often below 65%, and its visibility can be compromised in deep-lying SLNs, particularly in obese patients [100,101].

Radioactive tracer detection using a gamma-ray probe offers superior identification rates, reaching up to 93%, with effective penetration to depths beyond 50 mm [100]. Despite this advantage, it carries inherent disadvantages, including high background signals, lack of depth feedback, high costs, and radiation-related risks [102].

In recent years, near-infrared fluorescence detection using indocyanine green (ICG) has emerged as a promising alternative. ICG-based imaging demonstrates a high negative predictive value (>92%) and allows real-time visualization of lymphatic flow from the tumor to draining LNs [103]. ICG mapping can be performed either preoperatively via endoscopic injection into the submucosa or intraoperatively via laparoscopic subserosal injection, improving the accuracy of nodal dissection compared with traditional approaches that follow vascular anatomy [104]. Importantly, while ICG enhances detection rates and mapping accuracy across surgical approaches, ranging from 55% to nearly 100%, its specificity for malignant cells remains limited [105].

Beyond gastrointestinal cancer applications, mediastinal staging in non-small-cell lung cancer (NSCLC) exemplifies the broader implications of LN evaluation; despite negative findings on imaging, pathological upstaging (pN2) has been reported in up to 35% of patients with clinical N0 disease; information which was only provided after invasive nodal assessment [106]. EBUS has proven equally effective as mediastinoscopy in mediastinal staging, while offering a significantly lower complication rate, thereby representing a safer option for patients [107].

As illustrated in this brief review, intraoperative and SLNB guidance methods have evolved from simple dye-based techniques to sophisticated fluorescence and ultrasound-guided approaches. Each modality presents unique advantages and limitations, and its optimal use depends on tumor type, anatomical site, and available resources. Nevertheless, the lack of an intraoperative standardized, low-cost method, with high sensitivity and specificity for metastatic lymph nodes, continues to provide a suboptimal assessment, leaving occult metastases undetected.

#### 2.3.5. Postoperative Evaluation of Lymph Node Metastasis

Histopathological assessment remains the cornerstone of nodal evaluation for postoperative staging in CRC. It plays an important role in identifying residual disease, clarifying or confirming nodal status, and provides a guide for additional treatment modalities. However, its sensitivity is limited by methodological constraints. Manual palpation of surgical specimens, the standard technique for LN retrieval, may fail to identify smaller nodes, which can account for nearly half of all metastases [108]. To address this, intra-arterial methylene blue is injected into ex vivo colorectal cancer specimens [109]. Meta-analyses confirm that methylene blue significantly increases overall nodal yield and reduces the proportion of cases with inadequate sampling, thereby enhancing staging accuracy [110].

Beyond nodal yield, determining the presence of lympho-vascular invasion (LVI) is a critical determinant of risk stratification. Conventional hematoxylin and eosin (HE) staining often struggles to differentiate between lymphatic and vascular invasion, limiting interobserver agreement. The use of additional stains, such as D2-40 for lymphatic invasion and elastic stains like Victoria blue or Elastica van Gieson for vascular invasion, substantially improves diagnostic precision [111]. Evidence shows that additional staining methods have a higher diagnostic odds ratio for predicting nodal metastases compared to HE alone (6.0 vs. 2.7), enhancing both reproducibility and prognostic accuracy [112,113]. These findings suggest that supplementary staining techniques should be integrated into routine pathology workflows.

Altogether, postoperative staging strategies highlight the need for methodological rigor and technological innovation. Optimizing nodal harvest, incorporating advanced histological stains, and exploring the role of minimally invasive staging tools could significantly reduce the risk of under-staging and improve treatment individualization in colorectal cancer.

## 3. Future Directions

### 3.1. Towards Novel Diagnostic Approaches

One of the major challenges in CRC management is the lack of reproducible and accurate prediction tools for LNM. As previously detailed, current clinicopathological criteria may often lead to overtreatment, exposing many patients to unnecessary resections, while simultaneously failing to identify a subset of patients who would benefit from surgical intervention. Thus, there is an urgent need to establish strategies that minimize surgery-related morbidity and preserve quality of life without compromising oncological outcomes [114].

To bridge the gap between experimental designs and clinical implementation, several prospective studies and registry initiatives are currently evaluating advanced non-invasive nodal staging tools. Clinical trial cohorts are increasingly shifting focus toward verifying the accuracy of radiomics-driven algorithms and multi-center func-tional imaging protocols in real-world settings. For instance, prospective validation registries are currently evaluating the performance of high-resolution MRI coupled with texture analysis to preoperatively predict lymph node metastasis in early-stage (pT1-T2) colorectal cancer, aiming to safely reduce the rate of unnecessary radical resections. Additionally, pilot prospective evaluation trials assess the safety and diagnostic yield of real-time lymphatic contrast-enhanced ultrasound (CEUS) and fluorescence-guided sentinel lymph node mapping. These active clinical protocols represent critical regulatory pathways required to transition these advanced im-aging and computational platforms from bench to bedside.

SLN biopsy remains the standard for confirming LNM, yet it faces inherent limitations. A central challenge is that no single technique currently integrates preoperative localization, intraoperative guidance, real-time imaging, and safety into a single tool. Moreover, conventional preoperative methods lack real-time capabilities, creating a disconnect during surgery that reduces precision. Proof-of-principle devices combining ultrasound (US), photoacoustic, and fluorescence imaging suggest that real-time, multimodal guidance could improve SLN identification while reducing incision length and surgical morbidity, but malignant specificity remains the key bottleneck [96].

At present, no clinically available method currently enables whole-node, three-dimensional assessment of metastatic involvement prior to histopathology. Conventional pathology relies on microscopic examination of selected sections, with the risk of overlooking small metastatic foci outside the sampled planes [115]. These limitations of conventional histopathology are not restricted to a single institution but reflect a broader, systemic challenge in oncologic practice. Variability in nodal retrieval, processing techniques, and resource availability across centers further contributes to inconsistencies in staging accuracy. In parallel, artificial intelligence (AI) is increasingly used to reduce variability and support decision-making, including models operating on whole-slide imaging and clinicopathological predictors to refine risk assessment after endoscopic therapy [116], as well as emerging approaches that leverage preoperative radiologic staging data, although many studies remain limited by methodological bias and lack of robust external validation [117]. Beyond CRC, recent large-scale ultrasound AI systems have demonstrated that multimodal learning and explainability (e.g., attention-based fusion and risk heatmaps) can reach high diagnostic performance for nodal metastasis prediction, illustrating design patterns that are likely transferable to CRC when paired with appropriate acquisition and ground truth strategies [118,119,120,121]. Functional ultrasound variants such as lymphatic contrast-enhanced ultrasound also indicate that real-time, radiation-free SLN evaluation is feasible in prospective settings, providing complementary signals that may synergize with microstructural quantitative ultrasound readouts [122].

### 3.2. Reimagining Nodal Status with High-Frequency Ultrasound

High-resolution imaging modalities, particularly high-frequency quantitative ultrasound (HF-QUS), provide a promising alternative to redefine nodal assessment. Unlike standard B-mode ultrasound, HF-QUS provides quantitative estimates of microstructural tissue properties and enables 2D/3D scanning of the entire LN volume, independently of operator interpretation, offering the potential to detect LNM in real time [121]. This technique could transform nodal staging by providing a reliable, rapid, and noninvasive assessment before histological processing, thereby guiding both surgical and pathological decision-making. In this context, HF-QUS has already demonstrated high diagnostic accuracy in CRC patients, with sensitivity and specificity exceeding 85% for detecting LNM. Notably, this method successfully identified suspicious nodes with both diffuse metastases and micrometastatic foci [120].

Despite these promising diagnostic figures, several technical limitations and barriers to widespread clinical implementation must be acknowledged to provide a realistic translational perspective. At operating frequencies near or exceeding 33 MHz, acoustic attenuation increases dramatically, which strictly constrains the effective ultrasound penetration depth to a few millimeters. Consequently, transcutaneous or standard transrectal applications remain currently unfeasible for deep-seated mesenteric or retroperitoneal lymph nodes. Widespread clinical adoption is further bottlenecked by a steep operator learning curve and the lack of standardized training protocols. Clinicians and technicians must be specialized in interpreting high-frequency radiofrequency signals and parametric maps, which differ fundamentally from conventional B-mode imaging. Furthermore, the absence of standardized, commercially available hardware across institutions introduces significant acoustic calibration variability, necessitating robust phantom-based quality control and regulatory clearances before HF-QUS can be seamlessly integrated into routine surgical and pathological workflows (Table 4).

From a translational standpoint, HF-QUS is particularly attractive because it can be deployed ex vivo immediately after dissection (before fixation and sectioning) and, in principle, intraoperatively on exposed nodal basins. This creates a pathway toward selective intensification of pathology (targeted deeper sectioning or immunohistochemistry), and in the long term, toward biology-informed nodal staging strategies that may reduce unnecessary dissections while protecting oncologic safety [123,124]. It is important to emphasize that the proposed approach is not intended to replace conventional histopathological evaluation, which remains the gold standard for lymph node assessment, but rather to complement it. While postoperative pathology can be performed rapidly in some centers, it is inherently limited by sampling strategies, as only selected sections of each lymph node are examined. Consequently, small metastatic foci or micrometastases may be missed if they are not included in the analyzed planes.

This technology also offers the possibility of developing operator-independent scoring systems for malignancy prediction, enabling selective biopsy of suspicious nodes and cost reduction by limiting unnecessary sampling. Such methods could provide an alternative to SLNB, integrating prognosis and staging while informing personalized therapeutic strategies.

### 3.3. Future Perspectives: AI with 33 MHz HF-QUS in 2D/3D for Non-Invasive Nodal Assessment

The next major technological advance is the integration of HF-QUS (including ~33 MHz systems) with modern AI pipelines built for volumetric data. HF-US at these frequencies can resolve fine-scale acoustic heterogeneity, enabling quantitative parametric maps (e.g., scatterer size–related features and envelope-statistics parameters) that reflect stromal remodeling, tumor cellularity, necrosis, and immune–stromal architecture. However, the same frequency regime imposes practical constraints, most notably limited penetration depth, making early clinical impact most realistic in ex vivo scanning and intraoperative “point-of-care” assessment of accessible nodes.

**Figure 1:**
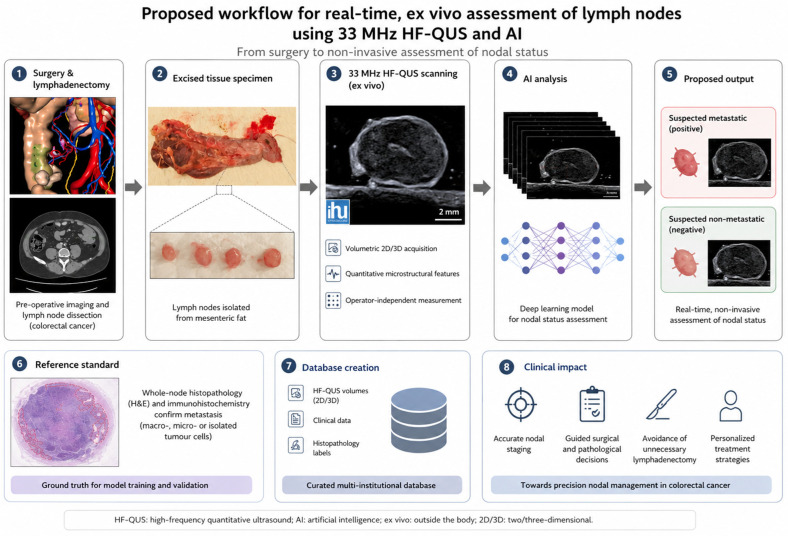
Proposed workflow for real-time, ex-vivo assesment of LN using 33MHz HF-QUS and AI.

A clinically deployable workflow is increasingly conceivable: rapid 2D sweeps or motorized/freehand 3D acquisitions reconstruct the LN volume; automated segmentation delineates nodal boundaries; RF-based estimators generate parametric maps; and a 3D model produces voxel-wise malignancy probabilities, along with uncertainty maps displayed as interpretable overlays aligned to B-mode. The key methodological opportunity is that HF-QUS naturally yields multi-channel, physics-linked representations (B-mode + parametric maps + potentially raw RF patches), which are well suited to multimodal deep learning and can be trained to be both accurate and explainable. In settings where voxel-level labels are unavailable, weakly supervised strategies (e.g., multiple-instance learning using node-level ground truth) can learn regional predictors, while histology-informed registration can progressively refine spatial supervision as whole-node reconstruction pipelines mature (Figure 1).

However, translating this conceptual vision into a clinically deployable pipeline re-quires anchoring it within concrete, high-performance computational frameworks that have already demonstrated feasibility in advanced medical image analysis. To over-come the limitations of generic AI descriptions, recent methodological breakthroughs in deep learning (DL) provide the exact technical roadmap needed for high-accuracy tumor staging. As systematically detailed in recent comprehensive surveys on complex oncological image analysis—such as advanced DL applications in bone tumor charac-terization—medical image processing has evolved significantly beyond basic classifica-tion tasks [125]. Current state-of-the-art frameworks primarily utilize Convolutional Neural Networks (CNNs), Vision Transformers (ViTs), and hybrid architectures [125]. Within these paradigms, U-Net frameworks serve as the standard for precise tissue and lesion segmentation, while You Only Look Once (YOLO) frameworks are widely adopted for real-time object detection and localization [125].

These identical DL architectures can be directly adapted for CRC lymph node metastasis detection. Specifically, hybrid ViT-CNN models can be trained on the mul-ti-channel, physics-linked representations of HF-QUS radiofrequency data to capture both local structural acoustic heterogeneity (via CNN convolutional layers) and global contextual features of the nodal architecture (via ViT self-attention mechanisms). Concurrently, U-Net-based segmentations can automatically delineate the nodal cortex and fatty hilum, allowing algorithms to detect micrometastatic effacements or tu-mor-induced angioarchitecture alterations that elude the human eye.

Morphological and microenvironment-related imaging features, however, are insufficient to fully capture the complex biological heterogeneity of CRC dissemination detailed in previous sections. The true paradigm shift relies on multimodal learning—the computation-al fusion of highly heterogeneous data sources to maximize predictive performance. The technical feasibility of this approach has been demonstrated by recent frameworks capable of integrating molecular sequences, similarity networks, and interaction graphs [126]. By leveraging pre-trained models combined with Heterogeneous Graph Neural Networks (HGNNs) and dynamic attention mechanisms, these architectures dynamically weigh different data modalities based on their contextual relevance, significantly outperforming existing predictive methods [126].

In the context of CRC staging, this multimodal paradigm can be directly translated into a clinical pipeline by fusing advanced imaging data (2D/3D pixel matrices from MRI and CT, alongside raw HF-QUS radiofrequency signals) with tabular clinical variables (such as patient risk factors, T-stage, and tumor topography) and high-dimensional molecular profiles. By processing genomic or transcriptomic sequences through pre-trained transformers and interaction graphs, the model can capture critical biolog-ical drivers, including epithelial-mesenchymal transition (EMT) markers like HSP47 and IGF-1R, VEGF-C expression levels, and metabolic fatty acid oxidation (FAO) up-regulation pathways.

These diverse inputs are funneled into an HGNN where a dynamic attention mecha-nism evaluates the cross-talk between the tumor’s acoustic or radiologic features and its underlying molecular aggressiveness. By transitioning from a purely descriptive an-atomical assessment to this evidence-based roadmap, advanced AI image analysis and multimodal integration can overcome the sensitivity limitations that currently plague CRC nodal staging, transforming a conceptual vision into a technically feasible implementation for precision, non-invasive care.

Generalizability will depend on standardization: QUS parameters are sensitive to calibration, attenuation compensation, and device-specific transfer functions. Multi-site deployment, therefore, needs reproducible acquisition protocols, phantom-based quality control, and algorithmic domain adaptation to prevent models from learning scanner signatures rather than biology. Encouragingly, the broader ultrasound AI literature shows that multicenter training and external validation can yield robust models for nodal metastasis prediction across institutions [120,121,122,123]. For CRC, the most credible path forward is to combine this implementation framework with the unique microstructural sensitivity of HF-QUS, targeting use cases where HF acquisition is feasible (ex vivo screening, intraoperative assessment, and potentially superficial nodal stations), and progressively expanding indications as hardware and acquisition strategies evolve.

Finally, multimodal ultrasound is likely to be additive rather than competitive. HF-QUS microstructure contrasts (scatterer/envelope statistics) can be complemented by vascular and lymphatic function signals (Doppler, contrast-enhanced ultrasound, lymphatic CEUS), and fused within a single predictive model. Such integration could improve sensitivity to early metastatic remodeling while maintaining specificity, ultimately converging on a pragmatic clinical promise: faster, more reproducible, whole-node assessment that triages pathology workload and supports more personalized nodal management in colorectal cancer [118,119,127].

## 4. Strengths and Limitations of the Review

This comprehensive review possesses distinct strengths, notably its deeply interdisci-plinary approach that successfully bridges basic tumor biology, advanced clinical deci-sion-making, and cutting-edge imaging innovations—such as HF-QUS and AI multi-modal frameworks. However, certain limitations must be acknowledged, the main limitation being the highly exploratory and non-standardized nature of the emerging diagnostic technologies discussed, which currently lack large-scale, multicenter clinical validation cohorts.

## 5. Conclusions

Lymphatic metastasis is a complex phenomenon regulated by cellular, molecular, and microenvironmental interactions. Accurate staging of LNM remains one of the greatest challenges in the management of CRC. Current strategies, heavily reliant on surgical resection and histopathology, often lead to overtreatment or understaging, highlighting a critical unmet need for reliable, non-invasive diagnostic alternatives. Therefore, the integration of advanced imaging, molecular biology, and AI technologies into clinical practice will provide new opportunities for earlier and more accurate identification of biologically relevant metastases, refine staging systems, and ultimately support risk-adapted, personalized therapeutic strategies.

HF-QUS has shown promising diagnostic accuracy in preliminary studies, offering operator-independent, real-time, and whole-volume LN assessment. While these innovations are not yet ready to supplant established guidelines, they lay the foundation for a new era of nodal staging in CRC, one in which invasive procedures may be progressively replaced by intelligent, non-invasive imaging, fostering better outcomes for patients and more sustainable healthcare delivery.

## Figures and Tables

**Table 1:** Biological mechanisms linked to nodal metastasis. Cellular and molecular drivers of lymphatic dissemination in CRC.

Category	Key Factors	Mechanism	Clinical Relevance
EMT	Snail, Twist, E-cadherin loss	Invasion/migration	Early dissemination
Lymphangiogenesis	VEGF-C/VEGFR-3	LEC proliferation	LN spread
ECM remodeling	MMPs, HSP47	Tissue invasion	Aggressiveness
Immune escape	MHC-I downregulation	Immune evasion	Metastatic survival
Metabolic adaptation	FAO, PPAR-α	LN colonization	Growth in LN niche

**Table 2:** Comparative summary of available preoperative imaging modalities for lymph node staging in colorectal cancer.

Modality	Sensitivity	Specificity	Key Advantages	Major Limitations	Clinical Applicability
**ERUS**	90–95%	70–90%	High resolution for the rectal wall; real-time evaluation of nodal morphology and cortical changes.	Highly operator-dependent; limited depth penetration; cannot visualize deeper pelvic or mesenteric nodes.	Standard tool for locoregional staging of rectal tumors.
**CT**	60–85%	70–90%	Rapid acquisition; excellent spatial resolution over large anatomical areas; ideal for retroperitoneal nodes.	Suboptimal accuracy when based on size criteria alone; radiation exposure; risks associated with contrast agents.	Routine initial staging for colon cancer and distant metastasis screening.
**MRI**	70–94%	67–90%	Superior soft-tissue contrast; multiplanar capabilities; DWI highlights restricted water diffusion.	Vulnerable to false positives due to inflammation; high costs; limited availability; longer acquisition times.	Gold standard for rectal cancer staging and mesorectal fascia involvement evaluation.
**PET-CT**	42.9–61%	83–87.7%	Combines functional and anatomical data; highlights high metabolic activity; excellent for distant recurrence.	Low spatial resolution; high rate of false negatives for small micrometastases (<5 mm); high cost.	Secondary tool for suspected systemic disease, staging discrepancies, or recurrence.
**PET-MRI**	~81%	~89%	Superior soft-tissue detail combined with metabolic data; excellent characterization of complex lesions.	Extremely high costs; very limited clinical availability; long acquisition times.	Emerging hybrid modality for advanced or doubtful pelvic staging scenarios.
**HF-QUS**	>85%	>85%	Real-time, operator-independent 2D/3D quantification of microstructural tissue and stromal properties.	Severely limited depth penetration at high frequencies (~33 MHz); requires exposed tissue or ex vivo specimens.	Promising for ex vivo surgical specimen screening and intraoperative assessment.

**Table 3:** Emerging and experimental imaging technologies. Next-generation approaches for nodal staging.

Technology	Principle	Advantage	Limitation	Stage
Texture CT/MRI	Radiomics	Quantitative features	Standardization issues	Early adoption
CEUS	Microbubbles	Real-time perfusion	Operator dependence	Clinical pilot
Photoacoustic imaging	Optical + ultrasound	High contrast	Limited depth	Experimental
Lymphoscintigraphy	Radiotracer mapping	Functional mapping	Low resolution	Established niche
HF-QUS	Microstructure analysis	Whole-node quantification	Penetration limits	Translational

**Table 4:** HF-QUS vs. conventional imaging. Comparative performance of HF-QUS vs. standard modalities.

Feature	HF-QUS	MRI	CT	PET-CT
Spatial resolution	High (microstructural)	Moderate	Low–moderate	Low
Functional info	Yes (microstructure)	Partial	No	Yes
Real-time	Yes	No	No	No
Operator dependence	Low	Moderate	Low	Low
Ability to detect micrometastasis	High	Low–moderate	Low	Low
Clinical readiness	Emerging	Established	Established	Established

## Data Availability

No new data were created or analyzed in this study. Data sharing is not applicable to this article.

## Abbreviations

The following abbreviations are used in this manuscript:

18F-FDG	Fluorodeoxyglucose (radiotracer used in PET)
2D	Two-Dimensional
3D	Three-Dimensional
AI	Artificial Intelligence
AQP(s)	Aquaporin(s)
CEUS	Contrast-Enhanced Ultrasound
CNNs	Convolutional Neural Networks
CRC	Colorectal Cancer
CT	Computed Tomography
D2-40	Immunohistochemical marker for lymphatic vessels
DL	Deep learning
DWI	Diffusion-Weighted Imaging
EBUS	Endobronchial Ultrasound
ECM	Extracellular Matrix
EGF	Epidermal Growth Factor
ENE	Extranodal Extension
ERUS	Endorectal Ultrasound
ESAM	Endothelial-Specific Adhesion Molecule
FAO	Fatty Acid Oxidation
FASN	Fatty Acid Synthase
FGF-2	Fibroblast Growth Factor-2
FNAB	Fine-Needle Aspiration Biopsy
HF-QUS	High-Frequency Quantitative Ultrasound
HE	Hematoxylin & Eosin
HGF	Hepatocyte Growth Factor
HSP47	Heat Shock Protein 47
ICG	Indocyanine Green
IFP	Interstitial Fluid Pressure
IGF-1	Insulin-Like Growth Factor-1
IGF-1R	Insulin-Like Growth Factor-1 Receptor
LNs	Lymph Nodes
LNM	Lymph Node Metastasis
LPA	Lysophosphatidic Acid
LEC(s)	Lymphatic Endothelial Cell(s)
LVI	Lympho-Vascular Invasion
MHC	Major Histocompatibility Complex
MMP	Matrix Metalloproteinase
MRI	Magnetic Resonance Imaging
NSCLC	Non-Small-Cell Lung Cancer
PDGF-BB	Platelet-Derived Growth Factor-BB
PET–CT	Positron Emission Tomography–Computed Tomography
PET–MRI	Positron Emission Tomography–Magnetic Resonance Imaging
PMN	Premetastatic Niche
PPAR-α	Peroxisome Proliferator–Activated Receptor Alpha
QUS	Quantitative Ultrasound
S1P	Sphingosine-1-Phosphate
SLN	Sentinel Lymph Node
SLNB	Sentinel Lymph Node Biopsy
TD(s)	Tumor Deposit(s)
TNM	Tumor, Node, Metastasis staging system
US	Ultrasound
VEGF-C	Vascular Endothelial Growth Factor-C
VEGFR-3	Vascular Endothelial Growth Factor Receptor-3
VGSC(s)	Voltage-Gated Sodium Channel(s)
ViTs	Vision Transformers
